# Modified linear regression predicts drug-target interactions accurately

**DOI:** 10.1371/journal.pone.0230726

**Published:** 2020-04-06

**Authors:** Krisztian Buza, Ladislav Peška, Júlia Koller

**Affiliations:** 1 Faculty of Informatics, ELTE – Eötvös Loránd University, Budapest, Hungary; 2 Center for the Study of Complexity, Babes-Bolyai University, Cluj Napoca, Romania; 3 Department of Software Engineering, Faculty of Mathematics and Physics, Charles University, Prague, Czech Republic; 4 Institute of Genomic Medicine and Rare Disorders, Semmelweis University, Budapest, Hungary; University of Ulm, GERMANY

## Abstract

State-of-the-art approaches for the prediction of drug–target interactions (DTI) are based on various techniques, such as matrix factorisation, restricted Boltzmann machines, network-based inference and bipartite local models (BLM). In this paper, we propose the framework of Asymmetric Loss Models (ALM) which is more consistent with the underlying chemical reality compared with conventional regression techniques. Furthermore, we propose to use an asymmetric loss model with BLM to predict drug–target interactions accurately. We evaluate our approach on publicly available real-world drug–target interaction datasets. The results show that our approach outperforms state-of-the-art DTI techniques, including recent versions of BLM.

## Introduction

When developing new drugs and identifying their side effects [1], pharmaceutical science relies on findings from related branches of science, including statistics and computer science. An essential step in this process is the identification of interactions between drugs and pharmacological targets. Although the existence of interactions can be reliably confirmed by *in vitro* binding assays, see e.g., [2–5], such methods are expensive and time consuming [6]. In order to address this bottleneck, computational approaches have been designed and implemented for the estimation of the probability of interactions. Therefore, most promising candidates for *in vitro* experiments may be selected based on *in silico* approaches.

The importance of drug–target interaction prediction is further emphasised by the costs of drug development. While estimates vary, they agree that it costs hundreds of millions of dollars to bring a new drug to the market, see e.g. [7] for an overview. Furthermore, the process may take more than 10 years in total.

Drug–target interaction prediction (DTI) techniques promise to reduce the aforementioned costs and time, and to support drug repositioning [8], i.e., the use of an existing medicine to treat a disease that has not been treated with that drug yet.

Drug repositioning is especially relevant for the treatment of rare diseases, including neurological disorders. While each of the rare diseases affect only few people, due to the large number of rare diseases, in total 6-8% of the entire population is affected by *one* of those diseases. This results in a paradox situation: although a significant fraction of the population is suffering from one of the rare diseases, it is economically irrational to develop new drugs for many of them. However, drug repositioning may potentially lead to breakthroughs in such cases.

In silico approaches for DTI include techniques based on docking simulations [9], ligand chemistry [10], text mining [11, 12] and machine learning. Text mining is inherently limited to the identification of entities and interactions that have already been documented, although the output of approaches based on text mining, i.e., the identified interactions, may serve as input data for other approaches, such as the ones based on machine learning. A serious limitation of docking simulations is that information about the three-dimensional structure of candidate drugs and targets is required. In many cases, e.g. for G-protein coupled receptors (GPCR) and ion channels, such information may not be available. Moreover, the performance of ligand-based approaches is known to decrease if only few ligands are known.

For the aforementioned reasons, state-of-the-art DTI techniques are based on machine learning [13–17]. Moreover, the increasing interest is also catalysed by the analogies between DTI and the well-studied recommendation tasks [18–20], which resulted in DTI approaches based on matrix factorisation [21–23]. Further recent DTI techniques are based on support vector regression [6], restricted Boltzmann machines [24], network-based inference [25, 26], decision lists [27], positive-unlabelled learning [16] and bipartite local models (BLM) [28]. Extensions of BLM include semi-supervised prediction [29], improved kernels [30], the incorporation of neighbour-based interaction-profiles [31] and hubness-aware regression [19].

Despite all the aforementioned efforts, accurate prediction of drug–target interactions still remained a challenge. In this paper, we propose a new regression technique for accurate DTI predictions. We use a novel loss function that reflects the needs of drug–target interaction better than wide-spread loss functions, such as mean squared error or logistic loss. Our generic framework of asymmetric loss models (ALM) works with various regressors. For simplicity, we instantiate ALM with linear regression which leads to *asymmetric loss linear regression* (ALLR). We propose to use this new regressor in BLM for drug–target interaction prediction. Note that ALM is substantially different from hubness-aware regressors that we used with BLM in our previous work [19]. As ALLR is a modified version of linear regression, we call our approach *Drug–Target Interaction Prediction with Modified Linear Regression*, or MOLIERE for short. We evaluate MOLIERE on publicly available real-world datasets and show that our approach outperforms state-of-the-art DTI techniques, including recent versions of BLM and the cases when conventional loss functions are used. Furthermore, we show that MOLIERE is able to predict medically relevant drug–target interactions that are not contained in the original datasets.

## Materials and methods

### Data

We used four publicly available real-world drug–target interaction datasets (Table 1), namely Enzyme, Ion Channel (IC), G-protein coupled receptors (GPCR), Nuclear Receptors (NR). The datasets are available at http://web.kuicr.kyoto-u.ac.jp/supp/yoshi/drugtarget/. These datasets have been used in various studies, such as [17, 19, 20, 22, 28, 29].

**Table 1:** Number of drugs, targets and interactions in the datasets used in our study.

Dataset	Drugs	Targets	Interactions
Enzyme	445	664	2926
Ion Channels (IC)	210	204	1476
G-protein coupled receptors (GPCR)	223	95	635
Nuclear Receptors (NR)	54	26	90

Each dataset contains an interaction matrix M between drugs and targets, a drug–drug similarity matrix $S^{D}$ and a target–target similarity matrix $S^{T}$. Similarities between targets were determined by the Smith-Waterman algorithm, see [17, 32] for details. Chemical structure similarities between drugs were computed using the SIMCOMP algorithm [33].

Each entry *m*_*i*,*j*_ of the interaction matrix M indicates whether the interaction between the *i*-th drug (denoted as *d*_*i*_) and *j*-th target (denoted as *t*_*j*_) is known:

$$\begin{array}{c} m_{i,j}=\{\begin{array}{cc} +1 & \text{if}\;\text{there}\;\text{is}\;\text{a}\;\text{known}\;\text{interaction}\;\text{between}\;d_{i}\;\text{and}\;t_{j}\\ -1 & \text{otherwise.} \end{array} \end{array}$$ (1)

Note that in case of these datasets, only the information about the *presence* of interactions is explicit, there is no explicit information about the *absence* of interactions. In particular, the semantics of *m*_*i*,*j*_ = −1 is that the corresponding drug *d*_*i*_ and target *t*_*j*_ *may* or *may not* interact. In fact, some of the drug–target pairs denoted as −1 actually interact, however, the interaction was unknown when these datasets were created, roughly 10 years ago. In order to allow for a fair comparison with other works in the literature, in our experiments reported in Tables 2–4, we used the original datasets without these “new” interactions.

**Table 2:** The performance of our approach, MOLIERE, compared with BLM and weighted profile (WP) with *k*_*d*_ = *k*_*t*_ = 5.

Dataset	Method	AUC	AUPR
Enzyme	MOLIERE	**0.990**	**0.924**
	BLM	0.973	0.841
	WP	0.955	0.868
Ion Channel	MOLIERE	**0.990**	**0.954**
	BLM	0.970	0.779
	WP	0.974	0.837
GPCR	MOLIERE	**0.974**	**0.837**
	BLM	0.953	0.667
	WP	0.943	0.648
NR	MOLIERE	**0.921**	**0.731**
	BLM	0.858	0.600
	WP	0.886	0.602

**Table 3:** The performance of our approach, MOLIERE, compared with the cases when using standard linear regression or logistic regression instead of the proposed regression technique.

Dataset	Method	AUC	AUPR
Enzyme	MOLIERE	**0.990**	**0.924**
	linear regression	0.980	0.924*
	logistic regression	0.971	0.870
Ion Channel	MOLIERE	**0.990**	**0.954**
	linear regression	0.982	0.952
	logistic regression	0.973	0.891
GPCR	MOLIERE	**0.974**	**0.837**
	linear regression	0.963	0.832
	logistic regression	0.917	0.752
NR	MOLIERE	**0.921**	**0.731**
	linear regression	0.888	0.724
	logistic regression	0.825	0.676

* In this case, the performance of MOLIERE with ALLR and MOLIERE with linear regression differs in the last digits that are not shown in the table.

**Table 4:** The performance of our approach, MOLIERE, compared with state-of-the-art DTI techniques. The best results are highlighted using bold font, the symbol •/∘ denotes whether the difference compared with the best approach is statistically significant (•) or not (∘).

Dataset	Method	AUC	AUPR
Enzyme	MOLIERE	**0.985**	**0.897**
	BLM-NII	0.966 •	0.628 •
	BRDTI	0.968 •	0.635 •
	HLM	0.966 •	0.832 •
	WNN-GIP	0.945 •	0.708 •
	NetLapRLS	0.959 •	0.784 •
Ion Channel	MOLIERE	**0.983**	**0.912**
	BLM-NII	0.960 •	0.626 •
	BRDTI	0.941 •	0.644 •
	HLM	0.980 •	0.867 •
	WNN-GIP	0.947 •	0.663 •
	NetLapRLS	0.966 •	0.827 •
GPCR	MOLIERE	**0.952**	**0.753**
	BLM-NII	0.929 •	0.387 •
	BRDTI	0.925 •	0.521 •
	HLM	0.947 ∘	0.686 •
	WNN-GIP	0.928 •	0.513 •
	NetLapRLS	0.910 •	0.580 •
NR	MOLIERE	**0.911**	**0.683**
	BLM-NII	0.879 •	0.543 •
	BRDTI	0.868 •	0.397 •
	HLM	0.864 •	0.576 •
	WNN-GIP	0.862 •	0.550 •
	NetLapRLS	0.810 •	0.428 •

In order to illustrate that our approach is indeed able to predict unknown interactions, we show that using the original data, we could predict many of those interactions that have been discovered meanwhile (Tables 5–7).

**Table 5:** Top 20 new interactions predicted by our approach, MOLIERE, on the Enzyme dataset. As additional information, we provide whether the interaction is validated (★) or not (−), and if it was predicted by other DTI techniques.

	Drug	Target	Val.	Also predicted by
1	D00542	hsa1571	★	BLM-NII, HLM, NetLapRLS, WNN-GIP, BRDTI
2	D00528	hsa1549	★	BLM-NII, HLM, NetLapRLS, WNN-GIP
3	D00437	hsa1559	★	BLM-NII, HLM, NetLapRLS, WNN-GIP, BRDTI
4	D00097	hsa5743	★	BLM-NII, NetLapRLS, BRDTI
5	D00139	hsa1543	★	BLM-NII, HLM, NetLapRLS, WNN-GIP
6	D03670	hsa1579	−	BLM-NII, HLM, NetLapRLS, WNN-GIP
7	D00574	hsa1589	★	HLM, NetLapRLS, WNN-GIP
8	D03670	hsa9420	−	HLM, NetLapRLS, WNN-GIP
9	D00437	hsa1585	★	BLM-NII, HLM
10	D03670	hsa51004	−	NetLapRLS
11	D03670	hsa51302	−	HLM, NetLapRLS
12	D00410	hsa1543	★	BLM-NII, HLM, NetLapRLS, WNN-GIP
13	D00449	hsa5742	−	HLM
14	D00410	hsa1583	★	HLM, NetLapRLS
15	D00947	hsa4129	−	−
16	D03670	hsa4353	★	−
17	D05458	hsa4128	★	HLM
18	D00043	hsa1990	★	BLM-NII, WNN-GIP
19	D00043	hsa1991	★	BRDTI
20	D00691	hsa5152	−	HLM, NetLapRLS

**Table 6:** Top 20 new interactions predicted by our approach, MOLIERE, on the GPCR dataset. As additional information, we provide whether the interaction is validated (★) or not (−), and if it was predicted by other DTI techniques.

	Drug	Target	Val.	Also predicted by
1	D00283	hsa:1814	★	BLM-NII, HLM, NetLapRLS, WNN-GIP
2	D04625	hsa:154	★	BLM-NII, HLM, BRDTI
3	D02358	hsa:154	★	BLM-NII, HLM, BRDTI
4	D02614	hsa:154	−	BLM-NII, HLM, BRDTI
5	D00110	hsa:1813	−	−
6	D00765	hsa:1128	★	BRDTI
7	D00760	hsa:1128	−	BRDTI
8	D00604	hsa:147	★	NetLapRLS
9	D02356	hsa:152	−	NetLapRLS
10	D00437	hsa:152	−	HLM, NetLapRLS, WNN-GIP
11	D02147	hsa:153	★	BLM-NII, HLM, BRDTI
12	D00095	hsa:155	★	BLM-NII, HLM
13	D02359	hsa:153	−	BLM-NII, BRDTI
14	D00397	hsa:1131	★	−
15	D00726	hsa:1129	★	−
16	D00255	hsa:152	★	HLM, NetLapRLS
17	D04375	hsa:151	★	−
18	D01103	hsa:1129	★	−
19	D05792	hsa:153	★	BRDTI
20	D01386	hsa:153	★	BRDTI

**Table 7:** Top 20 new interactions predicted by our approach, MOLIERE, on the IC dataset. As additional information, we provide whether the interaction is validated (★) or not (−), and if it was predicted by other DTI techniques.

	Drug	Target	Val.	Also predicted by
1	D00438	hsa:779	★	BLM-NII, HLM, BRDTI
2	D00294	hsa:10060	−	BLM-NII
3	D00552	hsa:6331	★	HLM, NetLapRLS
4	D00542	hsa:3736	−	−
5	D00649	hsa:8911	★	−
6	D00726	hsa:1138	−	BLM-NII, HLM, NetLapRLS, WNN-GIP
7	D03365	hsa:1137	★	HLM
8	D02098	hsa:8645	−	BLM-NII
9	D00538	hsa:6331	★	HLM, NetLapRLS
10	D00136	hsa:116443	−	−
11	D00349	hsa:773	−	BLM-NII, HLM
12	D00640	hsa:6336	★	HLM, NetLapRLS
13	D02356	hsa:6833	−	NetLapRLS
14	D00438	hsa:781	−	HLM
15	D00547	hsa:2570	−	BLM-NII, WNN-GIP
16	D00528	hsa:1080	−	BLM-NII
17	D00477	hsa:6336	★	BLM-NII, HLM, BRDTI
18	D00775	hsa:2898	★	BLM-NII
19	D00799	hsa:3782	−	BRDTI
20	D00638	hsa:8645	−	−

### Problem formulation

We define the Drug–Target Interaction Prediction problem as follows. We are given a set $D=\{d_{1},\ldots ,d_{n}\}$ of *n* drugs, a set $T=\{t_{1},\ldots ,t_{m}\}$ of *m* pharmaceutical targets, an *n* × *n* drug similarity matrix $S^{D}$, an *m* × *m* target similarity matrix $S^{T}$ and an *n* × *m* interaction matrix M. For some of the drug–target pairs the presence or absence of interaction is unknown (or simulated to be unknown in order to evaluate our approach). The task is to predict the likelihood of interaction for these unknown pairs.

At the first glance, the above DTI problem seems to be similar to the problems considered in the recommender systems community. Note, however, that most recommender techniques consider only the interactions (“ratings”) because even a few ratings are thought to be more informative than metadata, such as users’ similarity based on their demographic information [34]. In contrast, drug–drug and target–target similarities play an essential role in DTI.

### Bipartite local models

BLM considers DTI as a link prediction problem in bipartite graphs [28]. The vertices in one of the vertex classes correspond to drugs, whereas the vertices in the other vertex class correspond to targets. There is an edge *e*_*i*,*j*_ between drug *d*_*i*_ and target *t*_*j*_ if and only if *m*_*i*,*j*_ = 1.

The likelihood of unknown interactions is predicted as follows: we consider an unknown pair *u*_*i*,*j*_ = (*d*_*i*_, *t*_*j*_) and calculate the likelihood of interaction as the aggregate of two independent predictions.

**Fig 1:**
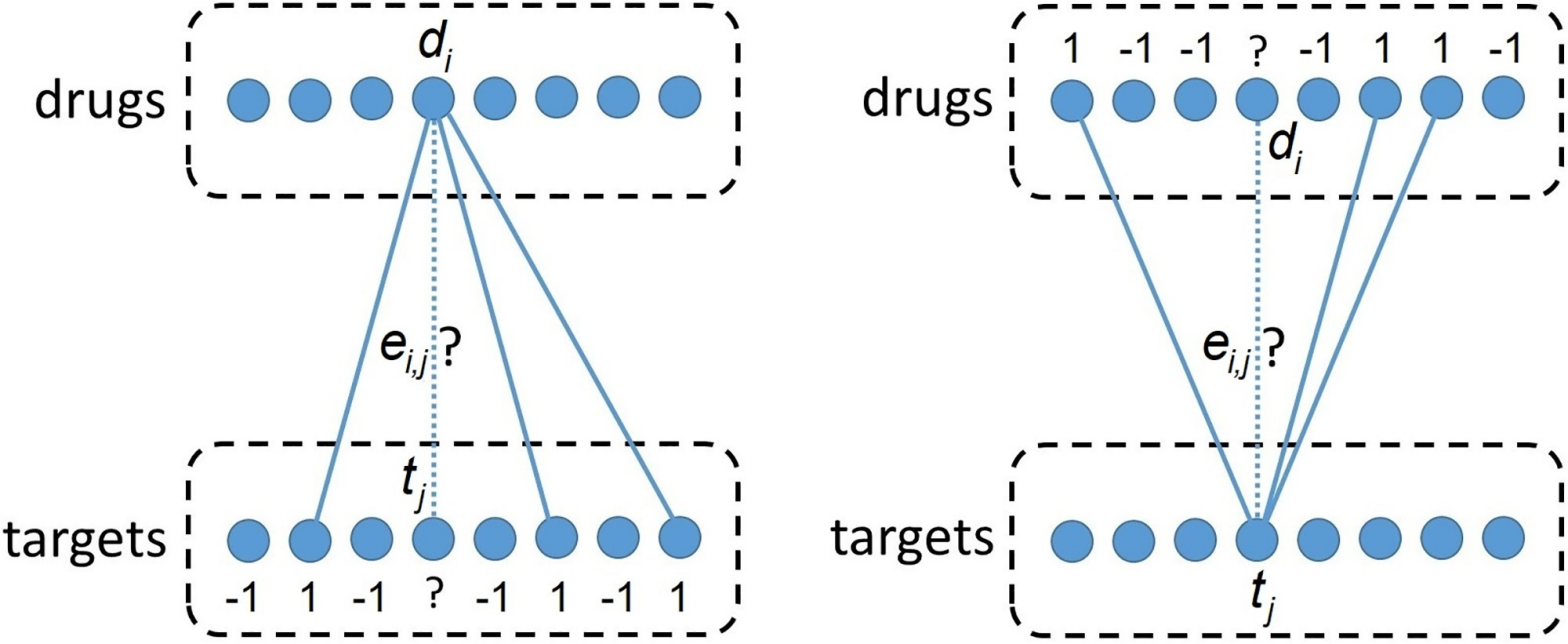
Predictions with BLM. Two predictions are calculated for the likelihood of each unknown interaction, i.e., for the presence of an edge *e*_*i*,*j*_. When calculating the first (second, respectively) prediction, targets (drugs, respectively) are labelled, and a local model is trained using these labels. Subsequently, the local model is used to predict the likelihood of the interaction between *d*_*i*_ and *t*_*j*_.

The first prediction, called *drug-centric prediction* (Fig 1, left panel), is based on the relations between *d*_*i*_ and the targets. Each target *t*_*k*_ (except *t*_*j*_) is labelled as “+ 1” or “−1” depending on *m*_*i*, *k*_. Then a model is trained to distinguish “+ 1”-labelled and “−1”-labelled targets. Subsequently, this model is applied to predict the likelihood of interaction for the unknown pair *u*_*i*,*j*_. This first prediction is denoted by ${\hat{y}}_{i,j}^{\prime }$. (When describing BLM, in accordance with our data, we assumed that only the information about the presence of an interaction is explicit, and therefore we train the model to distinguish known interacting pairs from pairs with unknown status. In contrast, if both *known interacting* and *known non-interacting* drug–target pairs are given, one may train the model using only the known interacting and known non-interacting pairs).

The second prediction, called *target-centric prediction*, ${\hat{y}}_{i,j}^{\prime \prime }$, is obtained similarly, but instead of considering the interactions of drug *d*_*i*_ and labelling the targets, the interactions of target *t*_*j*_ are considered and drugs are labelled (Fig 1, right panel). The models that make the first and second predictions are called *drug-centric* and *target-centric local models*.

In order to obtain the final prediction of BLM, we average the predictions of the aforementioned local models:

$$\begin{array}{c} {\hat{y}}_{i,j}=\frac{{\hat{y}}_{i,j}^{\prime }+{\hat{y}}_{i,j}^{\prime \prime }}{2} \end{array}$$ (2)

Note that instead of averaging, other aggregation functions, such as minimum or maximum are possible as well. According to our observations, our approach achieves most accurate results when the two predictions are averaged. However, the effect of the aggregation function can be considered as minor: when we repeated our experiments reported in Table 4 with *min* and *max* aggregation functions, we observed that our approach consistently outperformed its competitors for all the three aggregation functions. For example, on the GPCR dataset, our approach achieved an AUPR of 0.737 and 0.730 using *min* and *max* respectively, whereas we obtained an AUPR of 0.753 in case of averaging the two predictions.

BLM is a generic framework in which various regressors or classifiers can be used as local models. For example, Bleakley and Yamanishi [28] used support vector machines with a domain-specific kernel, whereas Buza and Peška used a hubness-aware regressor [19]. In our current work, we use BLM with asymmetric loss linear regression which will be described in the next section.

### Asymmetric loss models

Local models are the heart of BLM. Next, we propose a new regression technique that we use as a local model.

Given a regression model *f*_*θ*_ where *θ* is the vector of parameters, *f*_*θ*_ estimates the value of the target *y* for an instance *x* as $\hat{y}=f_{\theta }\left(x\right)$. In order to determine the appropriate parameter values θ*, usually, a loss function *L*_*D*_(*θ*) is minimised:

$$\begin{array}{c} \theta^{*}={\text{argmin}}_{\theta }\;L_{D}\left(\theta \right). \end{array}$$ (3)

Note that the actual value of *L*_*D*_(*θ*) depends both on the dataset *D* and parameters *θ*. However, once the dataset is fixed, in particular, while the model is being trained using a given training dataset *D*, the loss can be seen as a function of the parameter vector *θ*. Therefore, we aim at finding parameters θ* that minimise the loss. A wide-spread loss function is *mean squared errors*:

$$\begin{array}{c} L_{D}\left(\theta \right)=\frac{1}{\left|D\right|}\sum_{(x,y)\in D}{\left(f_{\theta }\left(x\right)-y\right)}^{2}, \end{array}$$ (4)

where |*D*| is the number of instances in *D*.

While the sum of squared errors is popular, we argue that in case of DTI, it is not fully consistent with the underlying chemical reality. In particular, binding energy may be different for various interactions. Consequently, in case of the presence of an interaction (*y* = + 1), we should not penalise a model that predicts a score that is higher than + 1. Similarly, in case of an unknown interaction (*y* = −1), we do not want to penalise a model that predicts a score that is lower than −1. Therefore, we propose an asymmetric loss function. First, we define the error of the model *f*_*θ*_ for a single prediction *f*_*θ*_(*x*), for instance *x* with label *y* as

$$\begin{array}{c} \text{err}\left(f_{\theta },x,y\right)=\{\begin{array}{cc} 0 & \text{if}\;f_{\theta }\left(x\right)>+1\;\text{and}\;y=+1\\ 0 & \text{if}\;f_{\theta }\left(x\right)<-1\;\text{and}\;y=-1\\ {\left(f_{\theta }\left(x\right)-y\right)}^{2} & \text{otherwise.} \end{array} \end{array}$$ (5)

We define *mean asymmetric loss (MAL)* as the mean of the above errors for all instances of the dataset *D*:

$$\begin{array}{c} MAL_{D}\left(\theta \right)=\frac{1}{\left|D\right|}\sum_{(x,y)\in D}\text{err}\left(f_{\theta },x,y\right). \end{array}$$ (6)

The above loss can be minimised with various optimisation techniques ranging from gradient-based methods to more advanced approaches, see e.g. [35]. For simplicity, we decided to use gradient descent. The partial derivative $\frac{\partial MAL_{D}\left(\theta \right)}{\partial \theta }$ of *MAL*_*D*_(*θ*) is:

$$\begin{array}{c} \frac{\partial MAL_{D}\left(\theta \right)}{\partial \theta }=\frac{1}{\left|D\right|}\sum_{(x,y)\in D}\frac{\partial \text{err}(f_{\theta },x,y)}{\partial \theta }, \end{array}$$ (7)

where

$$\begin{array}{c} \frac{\partial \text{err}(f_{\theta },x,y)}{\partial \theta }=\{\begin{array}{cc} 0 & \text{if}\;f_{\theta }\left(x\right)>+1\;\text{and}\;y=+1\\ 0 & \text{if}\;f_{\theta }\left(x\right)<-1\;\text{and}\;y=-1\\ 2(f_{\theta }\left(x\right)-y)\frac{\partial f_{\theta }\left(x\right)}{\partial \theta } & \text{otherwise.} \end{array} \end{array}$$ (8)

In case of linear regression where *x* = (*x*_1_, …, *x*_*k*_), *θ* = {*w*_0_, *w*_1_, …*w*_*k*_}, and the model is $f_{\theta }\left(x\right)=w_{0}+\sum_{i=1}^{k}w_{i}x_{i}$, the partial derivatives of err(*f*_*θ*_, *x*, *y*) according to *w*_*i*_, 1 ≤ *i* ≤ *k*, are

$$\begin{array}{c} \frac{\partial \text{err}(f_{\theta },x,y)}{\partial w_{i}}=\{\begin{array}{cc} 0 & \text{if}\;f_{\theta }\left(x\right)>+1\;\text{and}\;y=+1\\ 0 & \text{if}\;f_{\theta }\left(x\right)<-1\;\text{and}\;y=-1\\ 2(f_{\theta }\left(x\right)-y)x_{i} & \text{otherwise,} \end{array} \end{array}$$ (9)

while the partial derivative according to *w*_0_ is

$$\begin{array}{c} \frac{\partial \text{err}(f_{\theta },x,y)}{\partial w_{0}}=\{\begin{array}{cc} 0 & \text{if}\;f_{\theta }\left(x\right)>+1\;\text{and}\;y=+1\\ 0 & \text{if}\;f_{\theta }\left(x\right)<-1\;\text{and}\;y=-1\\ 2(f_{\theta }\left(x\right)-y) & \text{otherwise.} \end{array} \end{array}$$ (10)

**Fig 2:**
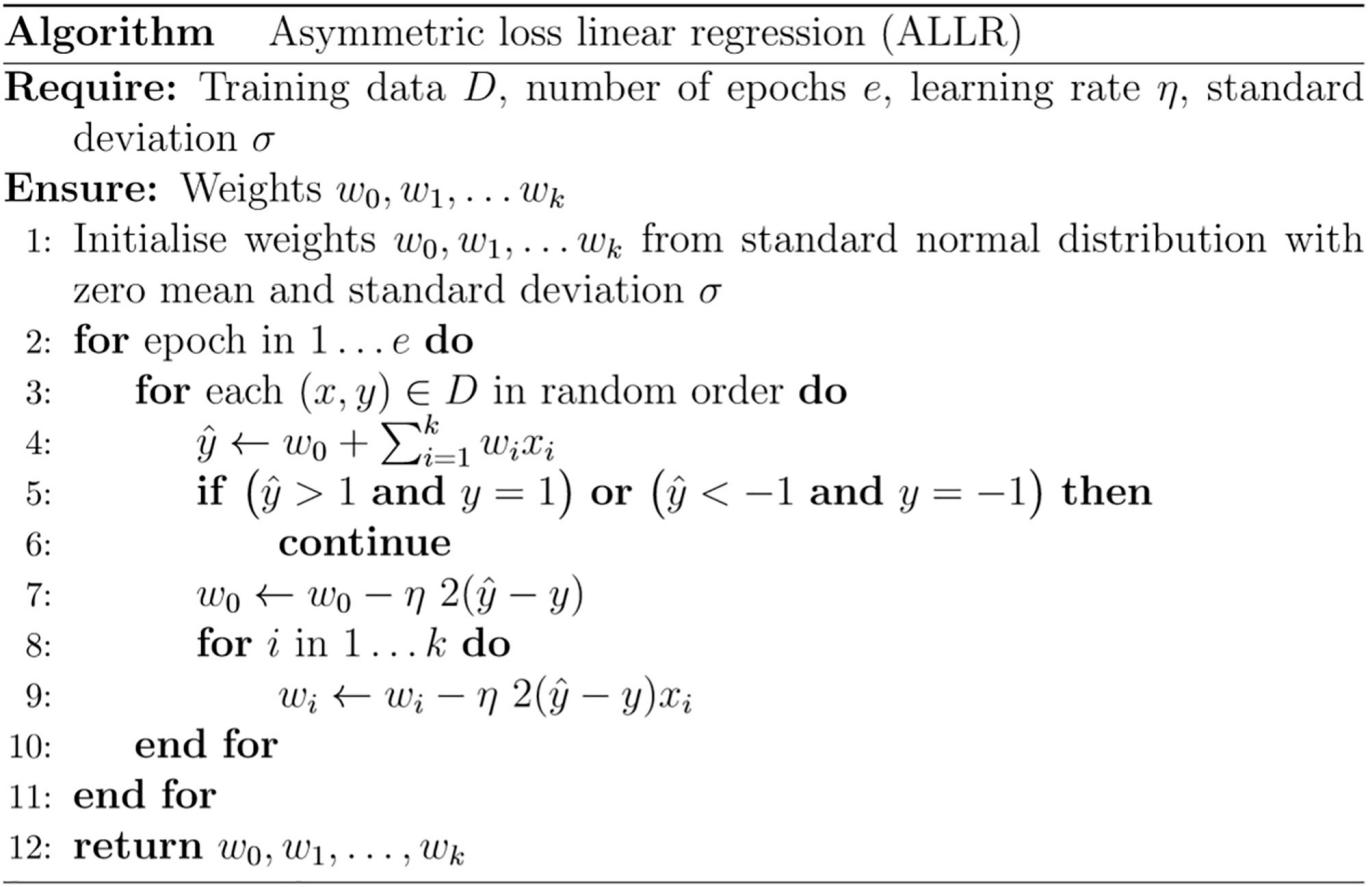
Pseudocode of asymmetric loss linear regression (ALLR).

We propose to use stochastic gradient descent to optimise *MAL*_*D*_. The pseudocode of the resulting asymmetric loss linear regression (ALLR) is shown in Fig 2.

### Weighted profile

One of the shortcomings of the BLM approach is that it does not handle the case of new drugs/targets. With *new drug* (or *new target*, respectively), we mean a drug *d* (target *t*) that does not have any known interaction in the (training) data. In such cases, BLM labels all targets (drugs) as “−1”, consequently, no reasonable local model can be learned. In order to alleviate this problem, we use the weighted profiles [17] of the most similar drugs/targets to obtain predictions for new drugs/targets.

Given a new drug *d*_*i*_, and a target *t*_*j*_, we predict the likelihood of the interaction between *d*_*i*_ and *t*_*j*_ as follows:

$$\begin{array}{c} {\hat{y}}_{i,j}^{\prime }=\frac{\sum_{r\in N^{D}\left(d_{i}\right)}m_{r,j}S_{i,r}^{D}}{\sum_{r\in N^{D}\left(d_{i}\right)}S_{i,r}^{D}}, \end{array}$$ (11)

where $N^{D}\left(d_{i}\right)$ denotes the set of indices of the *k*_*d*_ most similar drugs to *d*_*i*_ (not including *d*_*i*_ itself) based on the drug–drug similarities $S^{D}$.

The intuition behind Eq (11) is that similar drugs are likely to behave similarly in terms of their interaction with a given target. Therefore, drugs are weighed according to their similarity to the new drug *d*_*i*_ and we calculate the weighted average of the known interactions of other drugs with the same target.

The case of new targets is analogous. Given a new target *t*_*j*_ and a drug *d*_*i*_, the weighted profile approach can be used to calculate the prediction for the likelihood of the interaction between *d*_*i*_ and *t*_*j*_ as follows:

$$\begin{array}{c} {\hat{y}}_{i,j}^{\prime \prime }=\frac{\sum_{r\in N^{T}\left(t_{j}\right)}m_{i,r}S_{j,r}^{T}}{\sum_{r\in N^{T}\left(t_{j}\right)}S_{j,r}^{T}}, \end{array}$$ (12)

where $N^{T}\left(t_{j}\right)$ denotes the set of indices of the *k*_*t*_ most similar targets to *t*_*j*_ (not including *t*_*j*_ itself) based on the target-target similarities $S^{T}$.

Although the weighted profile approach is more general than BLM, in the sense that it can be used for new drugs/targets as well, the predictions of the weighted profile approach are usually less accurate than the predictions of BLM. Therefore, we use the weighted profile approach instead of BLM *only* in case of *new* drugs and targets.

### Our approach

We summarise our approach as follows. We use BLM for drug–target interaction prediction with the proposed asymmetric loss linear regression as local model in cases when the corresponding drug (target) has at least one known interaction and therefore the local model has at least one positive training instance. When initialising the parameters of ALLR, we use *σ* = 10^−8^. We train each ALLR model with a learning rate *η* = 10^−3^ for *e* = 100 epochs. According to our observations, ALLR is robust in the sense that the aforementioned settings allowed ALLR to converge to a model that outperformed other DTI techniques on all the examined datasets (see Table 4).

**Fig 3:**
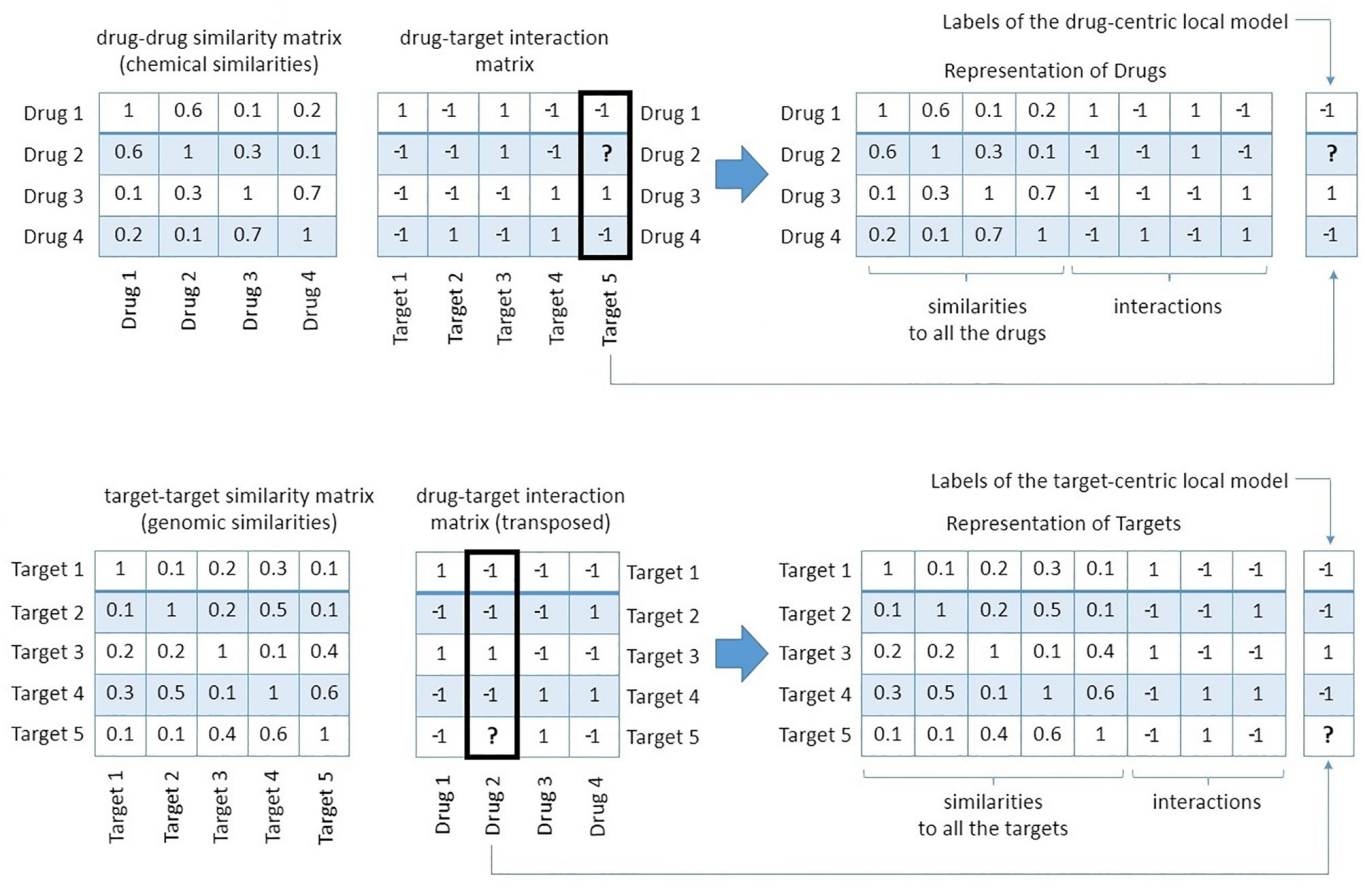
Representation of drugs and targets and labels of local models. In this example, the prediction is made for the interaction denoted by the question mark. Similarities with all drugs (targets, respectively) and interactions with all the targets (drugs), except the interactions with the target (drug) corresponding to the question mark, are used as features. The interactions with the target (drug) corresponding to the question mark are used as labels of the local models. Tables on the right represent the data used by the local model, i.e., ALLR in our case.

While predicting the interaction score between drug *d* and target *t* with ALLR, we represent each drug (target) as a vector of its similarities to all the drugs (targets) and its interactions, except the interactions with *t* (or *d* respectively), because the interactions with *d* (*t*) serve as labels for the local models, see Fig 3 for an illustration.

In case of new drugs (targets), we predict the likelihood of interactions using the weighted profile approach with *k*_*d*_ = *k*_*t*_ = 5.

## Results

### Comparison with baselines

As our approach, MOLIERE, is based on BLM, and uses weighted profile (WP) in case of new interactions, first, we compared the performance of MOLIERE to that of the original BLM and WP according to the widely used leave-one-interaction-out cross-validation protocol, see e.g. [28, 30, 31].

The predictions were evaluated both in terms of Area Under ROC Curve (AUC) and Area Under Precision-Recall Curve (AUPR). Table 2 shows that MOLIERE clearly outperforms both BLM and WP, both in terms of AUC and AUPR.

As the proposed asymmetric loss linear regression is a key component of MOLIERE, we examined the performance in case of alternative regression techniques, in particular we examined the cases when we use (a) standard linear regression and (b) logistic regression instead of ALLR. As expected, the proposed asymmetric loss linear regression indeed improves the quality of predictions, see Table 3.

### Comparison with recent DTI techniques

We compared MOLIERE with state-of-the-art drug–target interaction prediction techniques: two recent versions of BLM and three further prominent DTI approaches. The former include BLM with neighbour-based interaction-profile inferring (BLM-NII) [31] and hubness-aware regressors as local models (HLM) [19], while the later refer to net Laplacian regularized least squares (NetLapRLS) [29], a combination of weighted nearest neighbour and Gaussian interaction profile kernels (WNN-GIP) [36], and Bayesian Ranking for Drug–Target Interaction Prediction (BRDTI) [20].

Pahikkala et al. [37] pointed out that leave-one-out cross-validation may lead to overoptimistic results. Therefore, in this section, we used the interaction-based 5 × 5-fold cross-validation protocol, i.e., 5-fold cross-validation is repeated 5-times with different initial data splits. In each of the 5 × 5 rounds of the cross-validation, one fifth of the drug–target pairs were in the test data and AUC and AUPR values were calculated. The reported results are averaged values. In order to judge if the observed differences are statistically significant, we used paired *t*-test with significance threshold of *p* = 0.01.

Essential hyperparameters of BLM-NII, HLM, WNN-GIP, NetLapRLS and BRDTI were *learned* via grid-search in internal 5-fold cross-validation on the training data. For other hyperparameters that are not expected to have major impact on the results, we used default values according to the publication in which the approach was published.

In particular, for BLM-NII, as proposed by Mei et al. [31], the *max* function was used to generate final predictions and the weight *α* for the combination of structural and collaborative similarities was learned from {0.0, 0.1, …, 1.0}.

In case of HLM, according to [19], we performed experiments with *N* = 25 base prediction models, while the number of nearest neighbours for the local model EC*k*NN and the number of selected features, were learned from {3, 5, 7} and {10, 20, 50} respectively.

The decay hyperparameter of WNN-GIP was learned from {0.1, 0.2, …, 1.0} and the weight *α* for combination of structural and collaborative similarities was learned from {0.0, 0.1, …, 1.0}.

The hyperparameters of NetLapRLS (*β* = *β*_*drug*_ = *β*_*target*_ and *γ* = *γ*_*drug*_ = *γ*_*target*_), were learned from {10^−6^, 10^−5^, …, 10^2^}.

The content regularisation λ_*c*_ of BRDTI was learned from {0.1, 0.5, 0.9, 1.5}. The number of latent factors *f*, number of iterations, global regularisation λ_*g*_ and initial learning rate *η* were set to 100, 50, 0.01 and 0.1 respectively.

**Fig 4:**
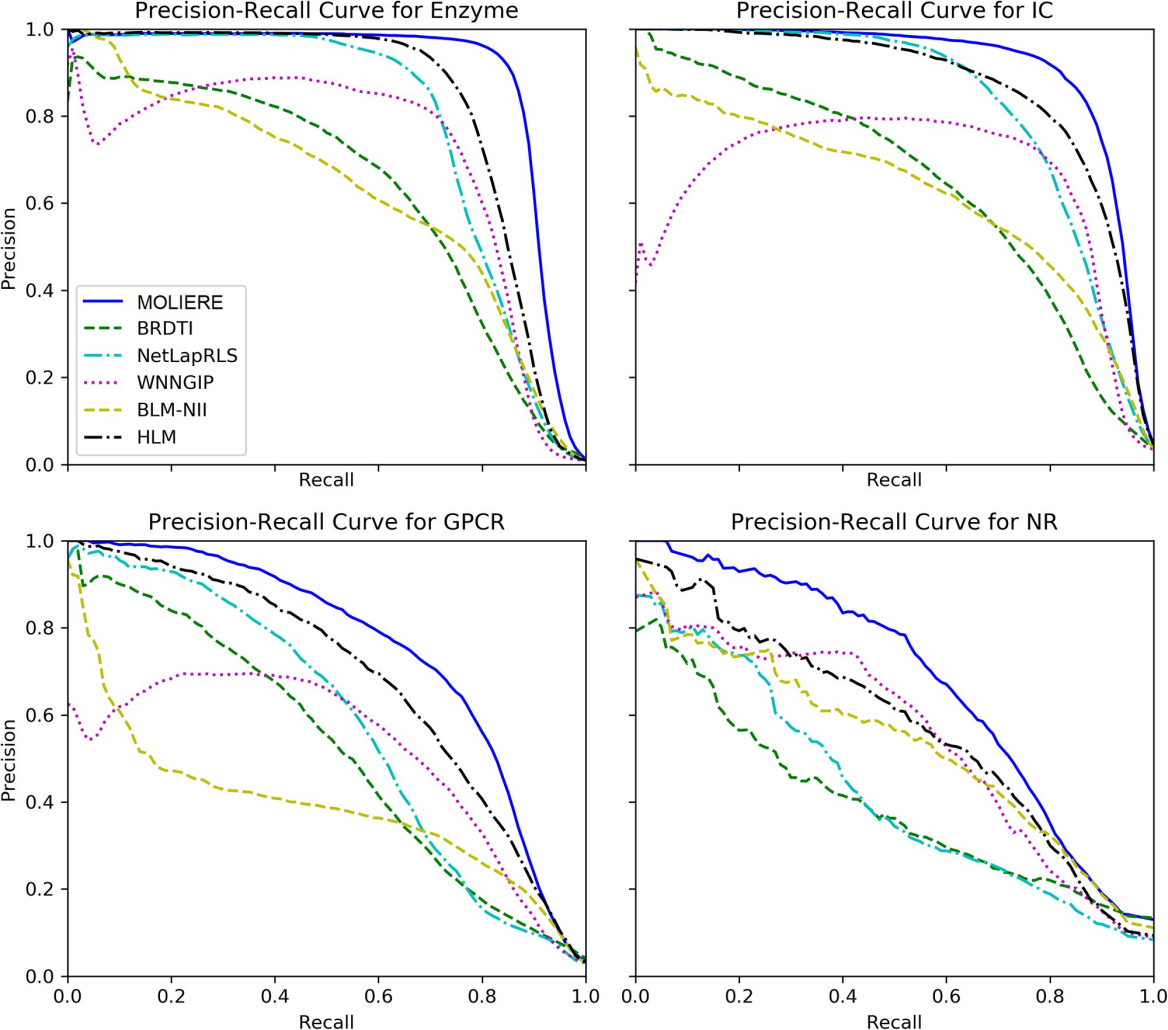
Precision-recall (PR) curves (averaged over the 5 × 5 folds of the cross-validation). As one can see, our approach, MOLIERE consistently outperforms its competitors: the PR-curve of MOLIERE is consistently above the PR-curves of its competitors.

The results are shown in Table 4 and Fig 4 which show the precision-recall curves for MOLIERE and its competitors. Our approach, MOLIERE, outperforms all the examined competitors in case of Enzyme, Ion Channel, GPCR and NR datasets, both in terms of AUC and AUPR. In the vast majority of the cases, the difference is statistically significant.

The results indicate that our approach, MOLIERE, is the overall best-performing approach out of the examined DTI techniques.

### Prediction of new interactions

In order to demonstrate that our approach is able to predict new interactions, we followed the same protocol as in [19], i.e., we trained our approach, MOLIERE, as well as its competitors, BLM-NII, HLM, NetLapRLS and WNN-GIP using all the interactions of the original datasets. As mentioned before, these datasets have been extracted roughly a decade ago and several additional interactions have been validated meanwhile. Our experiment aims to check whether these recently validated interactions can be predicted based on the original interactions.

In particular, we considered those drug–target pairs that have unknown interaction status in the original datasets. We ranked these pairs according to their predicted interaction scores. For simplicity, we use the term *predicted* interaction for the top-ranked 20 drug–target pairs. We say that a predicted interaction is *validated* if it is included in the current version of KEGG [38], DrugBank [39] or Matador [40].

The results are shown in Tables 5–7 for Enzyme, GPCR and IC datasets (for drug and target identifiers, see: http://www.kegg.jp/). As one can see, many of the predicted interactions are validated. We point out that some of the validated interactions were only predicted by our approach, especially in case of the GPCR dataset.

### MOLIERE for drug repurposing

In order to illustrate how our predictions may contribute to drug repurposing, we discuss some of the predicted interactions in more details.

First, we consider Diazoxide (KEGG ID: D00294) and “adenosine triphosphate binding cassette, sub-family C member 9” (ABCC9), also known as “sulfonylurea receptor 2” (SUR2) (KEGG ID: hsa:10060), i.e., the second predicted interaction listed in Table 7. According to KEGG, Diazoxide is an adenosine triphosphate (ATP) sensitive potassium channel opener. It opens potassium channel in beta cells of the pancreas and causes insulin secretion inhibition thus elevating blood sugar level. It is used in insulinoma [41] and congenital hyperinsulinism [42]. Diazoxide treatment can cause pulmonary hypertension and relax smooth muscle [43]. ABCC9 gene encodes a protein that is a subunit of an ATP sensitive potassium channel (ATP-binding cassette transporter) [44]. It is expressed in skeletal and heart muscle and in smooth muscles of the vasculature [44]. Mutation of the gene can cause dilated cardiomyopathy type 10 [45], and reduced cardiac stress adaptation [44]. ABCC9 knock-out mice showed elevated blood pressure and coronary artery vasospasm [46]. Other mutations of the ABCC9 gene (https://omim.org/entry/601439) can cause familial atrial fibrillation type 12 and hypertrichotic osteochondrodysplasia (Cantú syndrome) [47]. Diazoxide is not used in the treatment of these diseases. However, as Diazoxide can open the ATP sensitive potassium channel, it would be worth to examine the possible usage of Diazoxide in some ABCC9 gene defects where the transporter still can be activated to some extent.

Next, we consider the predicted interaction between Isradipine (KEGG ID: D00349) and “Calcium Voltage-Gated Channel Subunit Alpha1 A” (CACNA1A), also known as “spinocerebellar ataxia type 6” (SCA6) (KEGG ID: hsa:773), listed in the 11th line of Table 7. According to KEGG, Isradipine is an L type dihydropyridine calcium channel blocker that is used in hypertension. CACNA1A gene encodes the alpha-1A subunit of P/Q type voltage-dependent calcium channel. Mutations of this gene can cause spinocerebellar ataxia, early infantile epileptic encephalopathy, episodic ataxias, hemiplegic migraine and hemiconvulsion-hemiplegia-epilepsy syndrome. Some mutations of the gene increases the density of functional channels and their open probabilities in familial hemiplegic migraine [48]. Since our method takes into consideration the similarity of the two different calcium channels, it may be worth to try Isradipine inhibition of the P/Q type voltage-dependent calcium channel in experimental settings.

Finally, we consider the predicted interaction between Caffeine (KEGG ID: D00528) and Cystic fibrosis transmembrane conductance regulator (CFTR, KEGG ID: hsa:1080), listed in the 16th line of Table 7. Caffeine is a central nervous system stimulant, adenosine receptor antagonist and phosphodiesterase inhibitor (1). CFTR is a chloride channel that conducts chloride ions in lung, pancreas, liver, digestive tract and reproductive tract epithelial cell membranes. According to KEGG, gene mutations can cause cystic fibrosis (CF), hereditary pancreatitis and congenital bilateral absence of vas deferens. In rats caffeine intake increased CFTR chloride secretion in intestine [49]. Although, caffeine consumption is basically not recommended for CF patients, if some of the patients actually drink coffee, it would be interesting to compare their disease status with other CF patients not drinking coffee. Such a survey should be carefully designed in order to avoid biases. For example, the number of patients involved in the study should be large enough, one should take into account that people who have more sever disease may pay more attention to the health and lifestyle suggestions, while the type of mutations is also important.

## Conclusion

In this paper, we focused on drug–target interaction prediction and proposed a new method, MOLIERE for this task. Despite the fact that MOLIERE is a relatively simple approach, experiments on real-world datasets show that MOLIERE outperforms state-of-the-art DTI methods. By discussing some of the predictions in detail, we showed how our approach may lead to medically relevant hypothesis and support drug repositioning.

As mentioned, the DTI problem shares inherent characteristics with recommender systems tasks, therefore, we expect that MOLIERE will be adapted for recommendation tasks in the future. Furthermore, we point out that the proposed framework of asymmetric loss models is not limited to drug–target interaction prediction, but it may be useful in other cases where the class label is originally continuous (due to the underlying physical, chemical, biological phenomena), but it has been transformed to a binary label.

As for the limitations of our study, we note that our approach is not designed to predict interactions in case of new drugs/targets, i.e., for drugs/targets for which not even one interaction is known. In our current work, we assumed that only *few* new drugs/targets are considered, and we used the simple weighted profile approach in this case. Therefore, further methodical enhancements are required, if predictions are desired for new drugs/targets.
